# Inhibition of inflammatory response in human keratinocytes by magnetic nanoparticles functionalized with PBP10 peptide derived from the PIP2-binding site of human plasma gelsolin

**DOI:** 10.1186/s12951-019-0455-5

**Published:** 2019-02-02

**Authors:** Ewelina Piktel, Urszula Wnorowska, Mateusz Cieśluk, Piotr Deptula, Katarzyna Pogoda, Iwona Misztalewska-Turkowicz, Paulina Paprocka, Katarzyna Niemirowicz-Laskowska, Agnieszka Z. Wilczewska, Paul A. Janmey, Robert Bucki

**Affiliations:** 1 Department of Microbiological and Nanobiomedical Engineering, Medical University of Bialystok, Mickiewicza 2c, 15-222 Bialystok, Poland; 2 IInstitute of Nuclear Physics Polish Academy of Sciences, PL-31342 Krakow, Poland; 3 Institute of Chemistry, University of Białystok, Ciołkowskiego 1K, Białystok, Poland; 4 Department of Microbiology and Immunology, The Faculty of Medicine and Health Sciences of the Jan Kochanowski University in Kielce, Kielce, Poland; 5 Department of Physiology and Institute for Medicine and Engineering, University of Pennsylvania, Philadelphia, PA USA

**Keywords:** Gelsolin, Inflammation, Skin diseases, PBP10, Magnetic nanoparticles

## Abstract

**Background:**

Human plasma gelsolin (pGSN) is a multifunctional actin-binding protein involved in a variety of biological processes, including neutralization of pro-inflammatory molecules such as lipopolysaccharide (LPS) and lipoteichoic acid (LTA) and modulation of host inflammatory response. It was found that PBP10, a synthetic rhodamine B-conjugated peptide, based on the phosphoinositide-binding site of pGSN, exerts bactericidal activity against Gram-positive and Gram-negative bacteria, interacts specifically with LPS and LTA, and limits microbial-induced inflammatory effects. The therapeutic efficiency of PBP10 when immobilized on the surface of iron oxide-based magnetic nanoparticles was not evaluated, to date.

**Results:**

Using the human keratinocyte cell line HaCaT stimulated by bacterially-derived LPS and LTA as an in vitro model of bacterial infection, we examined the anti-inflammatory effects of nanosystems consisting of iron oxide-based magnetic nanoparticles with aminosilane (MNP@NH_2_) or gold shells (MNP@Au) functionalized by a set of peptides, derived from the phosphatidylinositol 4,5-bisphosphate (PIP2)-binding site of the human plasma protein gelsolin, which also binds LPS and LTA. Our results indicate that these nanosystems can kill both Gram-positive and Gram-negative bacteria and limit the production of inflammatory mediators, including nitric oxide (NO), reactive oxygen species (ROS), and interleukin-8 (IL-8) in the response to heat-killed microbes or extracted bacterial cell wall components. The nanoparticles possess the potential to improve therapeutic efficacy and are characterized by lower toxicity and improved hemocompatibility when compared to free peptides. Atomic force microscopy (AFM) showed that these PBP10-based nanosystems prevented changes in nanomechanical properties of cells that were otherwise stimulated by LPS.

**Conclusions:**

Neutralization of endotoxemia-mediated cellular effects by gelsolin-derived peptides and PBP10-containing nanosystems might be considered as potent therapeutic agents in the improved therapy of bacterial infections and microbial-induced inflammation.

## Background

Keratinocytes, comprising 95% of the human epidermis, are the first line of defense against external harmful agents and constitute an important part of the skin’s immune system [1]. In response to ultraviolet light, allergens, haptens, microbiological agents, and cytokines, keratinocytes express and release numerous immunomodulatory soluble mediators, including iNOS-derived NO, ROS, cytokines/chemokines, and prostaglandins. Those factors allow immune cells to enter the site of inflammation in the skin and contribute to the regulation of a variety of other physiological and pathological processes following an immune response [1, 2]. Although (i) cytokines and other epithelial cell-derived mediators participate in maintaining normal homeostatic mechanisms in the skin and (ii) inflammation itself is an important process to fight infections and promote wound healing, dysregulation of inflammatory responses and over-activation of pro-inflammatory mediators in the inflamed skin area lead to various inflammatory diseases [3]. Therefore, down-regulation of pro-inflammatory mediators and restoration of the physiological balance between pro- and anti-inflammatory factors, is an important strategy to modulate various inflammatory skin diseases [4]. In an era of constantly increasing bacterial drug resistance, therapeutic agents that neutralize bacterial pro-inflammatory factors, including lipopolysaccharide (LPS) and lipoteichoic acid (LTA) and thereby limit the inflammatory response resulting from activation of toll-like receptors (TLRs) in host cells, might represent an innovative approach for the treatment of bacterial infections, including skin and soft-tissue infections (SSTIs) or sepsis [5, 6]. Considering that SSTIs are the third most frequent cause of severe sepsis and septic shock [7], proper treatment of these infections is indispensable.

Plasma gelsolin (pGSN), an isoform of a highly conserved multifunctional human protein, gelsolin (GSN), apart from its role as an element of the extracellular actin scavenger system (EASS), is a potent modulator of inflammation-mediated cellular responses with the ability to diminish inflammatory reaction of the host [8, 9]. The beneficial effect of extracellular gelsolin is mediated primary via (i) selective interaction with cell wall-derived compounds i.e. LPS and LTA from Gram-negative to Gram-positive bacteria, respectively, (ii) competing with LPS-binding protein and (iii) prevention of endotoxin-mediated TLR activation [8]. pGSN binds a broad spectrum of bioactive compounds, including lysophosphatidic acid (LPA), sphingosine-1-phosphate (S1P), and platelet activating factor (PAF), enhancing the protective properties of GSN in inflammatory states [9–11]. Consequently, pGSN has been reported to have a beneficial effect in a variety of inflammatory-associated medical conditions, including sepsis, chronic inflammatory disorders such as chronic kidney disease, multiple sclerosis, rheumatoid arthritis, and inflammatory-mediated neurological disorders. pGSN levels have the potential to be used as a predictor of illness severity, recovery, the efficiency of treatment and/or clinical outcome [12, 13].

In addition to immunomodulatory properties of the intact GSN protein, it was found that a synthetic cell membrane-permeant rhodamine B (RhB)-conjugated peptide, based on the phosphatidylinositol 4,5-bisphosphate (PIP2)-binding site of gelsolin, (GSN 160-169 [rhodamine B-QRLFQVKGRR], denoted RhB-PBP10), interacts specifically with LPS and LTA and exerts strong bacterium-killing activity against both Gram-negative and Gram-positive bacteria due to strong resemblance to natural antimicrobial peptides by displaying net positive charge, short sequence and ability to cross cell membranes [14]. Fu et al. showed that RhB-PBP10 selectively inhibits granule mobilization and secretion of oxygen radicals in FPRL1-induced neutrophils [15]. A majority of the data suggesting a beneficial function of pGSN or RhB-PBP10 has been obtained using pure extracts of endotoxins and isolated bacterial strains in a non-growing environment and studies using more complex cell culture-based experiments are still very limited.

The rapid development of novel nanotechnology-based therapeutic strategies has provided new tools for treatment of infections, particularly those caused by drug-resistant pathogens and has created the possibility of overcoming limitations of conventional antibiotic therapy and improve the bioavailability of bioactive substances and their antimicrobial and immunomodulatory properties. Nanoscale materials, including synthetic biodegradable polymers such as chitosan or poly-lactic-co-glycolic acid (PLGA), polysaccharide or carbon dots-based nanoparticles have been extensively studied for their promising use in biomedical technology as immunostimulants and adjuvants promoting the immunomodulatory properties of other bioactive compounds [16, 17]. Among several kinds of nanomaterials, metal and metal oxide-based nanoparticles have potential use in modulation of inflammatory immune responses due to (i) limitation of inflammatory marker release, including ROS and nitric oxide in LPS-stimulated immune cells, (ii) ability to bind and remove endotoxins from the extracellular environment, (iii) modulation of gene expression and (iv) augmentation of immunomodulatory properties of other conjugated compounds [18–21]. The usefulness of nanoparticle-containing compounds was also confirmed in some in vivo models of LPS-induced pathology [22–24]. Our recent study of the bactericidal and immunomodulatory properties of 1,4-dihydropyridiyne (1,4-DHPs) derivatives, revealed that attachment of these bioactive compounds to aminosilane-coated iron oxide-based magnetic nanoparticles (MNP@NH_2_) significantly improves the immunomodulatory properties of 1,4-DHPs and inhibits the proinflammatory properties of bacterial cell wall components, which is a significant advance in creation of a novel class of multimodal agents for the treatment of life-threatening infections [25].

In this report, we characterize the immunomodulatory properties of a set of gelsolin-derived peptides based on the PIP2-binding site of gelsolin (i.e. GSN160-169) both in free form and conjugated with rhodamine B, and evaluate the impact of attachment of these bioactive peptides to aminosilane-coated and gold-decorated iron oxide-based magnetic nanoparticles on their ability to inhibit the bacterial-induced inflammatory responses in a cell culture-based model of skin infection. These studies indicate the enhancement of biocompatibility and improvement of biological features of the therapeutic peptides after their immobilization on magnetic nanocarriers when compared to their non-magnetic counterparts. We suggest that the increased anti-inflammatory properties of gelsolin-derived compounds after their immobilization on magnetic particles is related to improved cellular uptake and the properties of non-modified nanoparticles, particularly gold-decorated nanostructures to limit inflammatory response. This augmentation of peptide bioactivity suggests the possible application of MNP-based approaches in the development of improved anti-infectious therapeutic agents with combined anti-inflammatory functions that diminish the excessive inflammatory reaction of the host in the response to induction by bacterial-derived compounds.

## Materials and methods

### Peptides

The set of gelsolin-related peptides, based on the free non-conjugated sequence of GSN160-169 (QRLFQVKGRR, or PBP10) and PBP10 conjugated to rhodamine B (denoted with RhB- as a prefix to the peptide name) with or without a terminal cysteine (RhB-PBP10-Cys and RhB-PBP10, respectively) were synthesized and provided by Lipopharm.pl (Zblewo, Poland). According to HPLC analysis provided by the manufacturer, the purity of the synthesized peptides was > 98%.

### Bacterial products

Lipopolysaccharide from *Escherichia coli* O26:B6 and lipoteichoic acid from *Staphylococcus aureus* were purchased from Sigma Chemical Co. (St. Louis, Mo., USA). Stock solutions of LPS and LTA were prepared by suspending them in endotoxin-free water (Sigma Chemical Co.). Heat-killed *S. aureus* was prepared by boiling the bacteria for 7 min and then washing them three times with phosphate-buffered saline (PBS). The efficacy of the heat treatment was confirmed by culturing the bacteria overnight to ensure that there was no growth.

### Synthesis and physicochemical characterization of PBP10-containing nanosystems

Nanosystems used in this study were obtained using iron oxide-based magnetic nanoparticles with aminosilane (MNP@NH_2_) or gold shells (MNP@Au). The magnetic core of the nanocarriers was synthesized using a modification of the Massart method, which is based on the coprecipitation of hydrated iron chloride salts after addition of ammonium hydroxide (25%) [26]. Core–shell nanostructures with terminal propylamine groups and gold shells were obtained using Stöber and K-gold methods, respectively [27]. Following synthesis, all the nanoparticle samples were placed in an oven at 60 °C and dried into powder over 12 h. Physicochemical analysis of aminosilane- and gold-decorated nanoparticles were presented previously [27].

PBP10-containing nanosystems were obtained by non-covalent bonding including electrostatic interactions or chemisorption of thiol groups to the gold surface. Nanoparticles (MNP@NH_2_ or MNP@Au) were dispersed in PBS to obtain solutions of 10 mg/mL concentration. Then nanoparticles were diluted to the concentration of 1 mg/mL and were mixed with the appropriate amount (1:1 volume ratio) of PBP peptide or PBP modified by cysteine and rhodamine (1 mg/mL solutions in PBS buffer). Prepared solutions were incubated for 2 days (nucleation) and used for further investigations. In order to avoid the loss of agents during the preparation process, no further purification of nanosystems was performed. To evaluate the efficiency of peptide attachment, fluorescence of unbound PBP10 peptides in solutions after magnetic separation of nanosystems was measured.

Fourier transform infrared spectroscopy (FTIR) spectra were recorded using a Thermo Fisher Scientific Nicolet iN10 MX FTIR microscope. For this purpose, a 10 μL sample (1 mg/mL) was dropped on the surface of a glossy metal plate, and the solvent was left to evaporate. All spectra were collected in the 4000–800/cm range by co-adding 64 scans with a resolution of 4/cm. Data analysis was performed using OMNIC software (Thermo Fisher Scientific). Hydrodynamic diameters (DLS) were measured at 25 °C using a Zetasizer NanoZS (Malvern Instruments, Ltd, UK) with integrated 4 mW He–Ne laser, λ = 633 nm. Light scattering intensity was measured at 173° in case of all samples. The concentration was 1 mg/mL of nanosystems in PBS buffer solution. The zeta-potential measurements were carried out on the same Zetasizer NanoZS analyzer using the same solutions. All measurements were carried out at 25 °C using folded capillary cells (DTS 1060). Data were generated in the form of electrophoretic mobility, which was further converted to zeta potential by application of the Smoluchowki equation.

### Cell culture

Immortalized adult human skin keratinocytes cells (HaCaT) were grown in high-glucose Dulbecco’s Modified Eagle Medium (DMEM) supplemented with 10% fetal bovine serum (FBS), glutamine (2 mM/L), penicillin (50 U/mL) and streptomycin (50 µg/mL) and maintained at 37 °C in an atmosphere containing 5% CO_2_ with saturated humidity. After seeding, the cells were cultured until a confluence of ~ 85% was reached. All experiments were performed in serum-free conditions.

### Assessment of biocompatibility of analyzed nanosystems

The viability and metabolic activity of cells were measured using a microculture tetrazolium test (MTT; 3-(4,5-dimethylthiazol-2-yl)-2,5-diphenyltetrazolium bromide) as described previously [28]. HaCaT cells were seeded at a density of 5 × 10^4^ cells/well in transparent 96-well plates and cultured until ~ 85% confluence; after that medium was replaced with serum-free medium and cells were serum starved for another 12 h. To determine the non-toxic concentrations, the peptides and their magnetic derivatives in concentrations ranging from 1 to 50 µg/mL were added to each well and incubated for 24 h at 37 °C under 5% CO_2_. The optical density at 490 nm was assessed after 2 h incubation of plates with MTT solution (5 mg/mL) and the addition of formazan salt in dimethyl sulfoxide (DMSO). The absorbance value obtained in cultures of control cells (with solvents alone) was taken as 100%. The average of all the experiments is shown as cell viability percentage in comparison to the control.

### Quantification of hemolytic activity

The hemolytic activity of each peptide and its nanosystem was determined using a previously described method [29]. Briefly, fresh human red blood cells (RBCs) were collected and then centrifuged at 1000×*g* for 5 min at 4 °C. The erythrocytes were then washed three times with PBS (pH 7.2), resuspended (hematocrit ~ 5%) in PBS containing antibacterial agents at a concentration ranging from 0 to 50 µg/mL and incubated for 1 h at 37 °C. Intact erythrocytes were centrifuged at 1000×*g* for 5 min at 4 °C, and the supernatant was transferred to a 96-well microtiter plate. The release of hemoglobin was monitored by measuring the optical density at 570 nm (OD_570_). 100% hemolysis was taken from samples in which 1% Triton X-100 was added to disrupt all cell membranes. Minimum hemolytic concentrations (MHC10%) are defined as the concentration that causes hemolysis of 10% of RBCs.

### Antibacterial testing

To assess the bactericidal activity of PBP10-derived peptides and PBP10-containing nanosystems against *E. coli* and *S. aureus* bacterial strains, a killing assay based on counting colony forming units (CFU) was performed [30]. For this purpose, *E. coli* and *S. aureus* strains were grown to mid-log phase at 37 °C, re-suspended in PBS, brought to 10^8^ CFU/ml (which corresponds to 0.5 OD at a wavelength 600 nm) and diluted 1:100 to obtain sample containing approx. 10^6^ CFU/mL. Bacteria were then added to PBS containing different concentrations of PBP10, RhB-PBP10 and RhB-PBP10-Cys and their nanosystems at doses of 2, 5 and 10 µg/mL. After 1 h of incubation at 37 °C, the plates were transferred to ice and suspensions were diluted 10- to 1000-fold in PBS. Then, 10 μL aliquots were spotted on agar plates for overnight culture at 37 °C in order to determine CFUs.

### Nitric oxide assay

The nitric oxide assay was performed as described previously [31] and the quantity of nitrite in the culture medium was measured as an indicator of NO production. Amounts of nitrite, a stable metabolite of NO, were measured using the Griess reagent (1% sulfanilamide and 0.1% naphthylethylenediamine dihydrochloride in 2.5% phosphoric acid). Briefly, 5 × 10^4^ HaCaT cells were seeded in transparent 96-well plates and cultured until 85% confluence was reached. After that, cells were washed with PBS, and agents at final concentrations of 2, 5 and 10 µg/mL were added to each well. Simultaneously, keratinocytes were stimulated with 1 µg/mL LPS (*E. coli* 026:B6), 1 µg/mL LTA (from *Staphylococcus aureus)* or heat-inactivated suspensions of *E. coli* or *S. aureus* (~ 10^6^ CFU/mL) and incubated in serum-free medium for 24 h. Next, 50 µL of cell culture medium was mixed with 50 µL of Griess reagent, incubated at room temperature for 15 min, and the absorbance at 540 nm was measured in a microplate reader. Fresh culture medium was used as a blank in every experiment.

### ROS formation

The generation of reactive oxygen species from stimulated HaCaT cells was measured using 2′,7′-dichlorofluorescein diacetate (DFCH-DA) as the fluorescent probe. For this purpose, 5 × 10^4^ of HaCaT cells, cultured until ~ 85% confluence and incubated simultaneously with peptides or their magnetic derivatives at concentrations of 2, 5 and 10 µg/mL and with 1 µg/mL LPS/LTA or heat-inactivated bacteria for 24 h. Next, cells were washed twice with PBS, and DFCH-DA in PBS at the concentration of 20 µM was added. Fluorescence was measured for 90 min immediately after the addition of the dye at excitation/emission wavelengths of 488/535 nm.

### IL-8 release

In order to assess release of interleukin-8 (IL-8) from stimulated keratinocytes, 5 × 10^4^ HaCaT cells were treated with agents at concentrations of 2, 5 and 10 µg/mL and stimulated with 1 µg/mL LPS/LTA. The medium from LPS/LTA-treated cells was harvested after 24 h of incubation and the level of IL-8 was evaluated using an IL-8 Human ELISA Kit (Thermo Fisher Scientific).

### Atomic force microscopy (AFM)

The stiffness of cells after 24 h stimulation with 1 µg/mL LPS was measured by indentation using atomic force microscopy (Nanowizard 4 Bioscience AFM, JPK Instruments, Germany). Adenocarcinomic human alveolar basal epithelial cells (A549 ATCC^®^ CCL-185™) were cultured in high-glucose DMEM supplemented with 10% FBS at 37 °C with 5% CO_2_, placed on Ø 35 mm Petri dishes and allowed to attach and spread for 24 h before simultaneous stimulation with 1 µg/mL LPS (*E. coli* 026:B6) and treatment with 5 µg/mL of PBP10 or PBP10-based nanosystems. Immediately before the experiment, cells were placed in a CO_2_-independent buffer (Thermo Fisher Scientific, USA) to prevent changes in pH of the cellular environment during analysis. Elasticity measurements were taken with AFM working in force spectroscopy mode in liquid conditions, and cantilevers (ORC8, Bruker) with a spring constant of 0.1 N/m were used. From each tested cell, up to 64 force-indentation curves were collected in a grid of 8 × 8 pixels corresponding to a scan area of 10 × 10 µm with a maximal force of 2 nN. For indentation measurements, more than 1100 force-distance curves were recorded for each group from at least ten different cells. To determine the apparent Young’s modulus of different cells, force-indentation curves were fit to the Hertz contact model.

### Statistical analysis

The significance of differences was determined using the two-tailed Student’s *t* test. Statistical analyses were performed using Statistica 10 (StatSoft Inc, Tulsa, OK, USA). *p *< 0.05 was considered to be statistically significant. Results are the average of three to six individual measurements.

## Results

### Synthesis and physicochemical characterization of PBP10-containing nanosystems

**Fig. 1:**
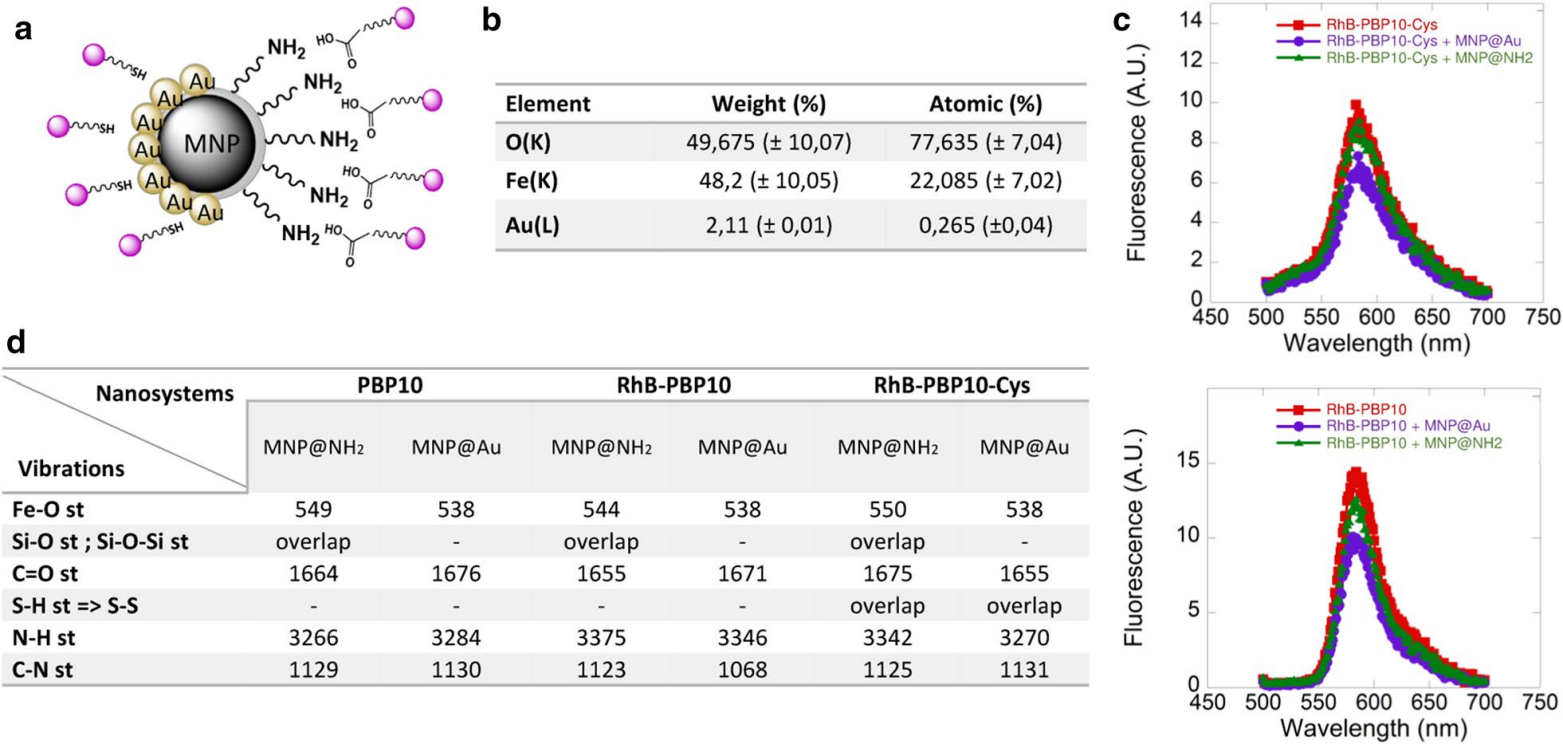
Physicochemical characterization of PBP10 peptides functionalized by gold- and aminosilane-coated magnetic nanoparticles. Schematic representation of MNP@NH2/MNP@Au-based nanosystems (**a**). Purple circles indicate gelsolin-derived peptides attached to the surface of MNPs. Elemental analysis of gold-decorated nanomaterials (**b**). Fluorescence spectra of RhB-PBP10 and RhB-PBP10-Cys peptides anchored to the surface of aminosilane- and gold-decorated nanomaterials (**c**). A comparison of the Fourier transform infrared spectra for PBP10, RhB-PBP10 and RhB-PBP10-Cys-based nanosystems (**d**)

In our study, synthesized nanosystems were obtained via physicochemical interaction between the surface of core–shell nanoparticles composed of an iron-oxide core and gold or aminosilane shells and the chemical groups of peptides. PBP10 peptides were anchored to nanoparticles structure via non-covalent interactions including electrostatic attractions or chemisorption of the thiol group to the gold surface (Fig. 1a). To confirm the presence of a gold surface on the nanoparticles, elemental analysis indicated that the gold content is ~ 2% of the nanostructure weight (Fig. 1b). To determine if PBP10 peptide derivatives were successfully immobilized on the core–shell surface fluorescent spectra were recorded. The similarity between the spectra of peptides in free and immobilized forms confirms that peptides became attached to the particles (Fig. 1c). To confirm that free, non-attached peptides do not persist in the MNP suspension, fluorescence-based measurements were performed. Results of fluorescence measurements for solutions after magnetic separation of MNP@PBP10-RhB and MNP@PBP10-RhB-Cys are presented in Table 1. As presented, PBP10-RhB peptide is immobilized on the surface of both MNP@Au and MNP@NH2 with nearly 100% efficiency, since after magnetic separation nearly non-detectable fluorescence is recorded. The amount of unbound PBP10-RhB-Cys peptide does not exceed 5%. Considering that doses of nanosystems used in our experiments are relatively low (i.e. 1–10 µg/mL), we assume that the concentration of unbound peptide was too small to affect collected data.

**Table 1:** Fluorescence of solutions obtained after magnetic separation of MNP@RhB-PBP10 and MNP@RhB-PBP10-Cys suspensions

	Fluorescence intensity (A.U.)	% of remaining peptide
RhB-PBP10 [1 mg/mL]	324.1	n/a
RhB-PBP10-Cys [1 mg/mL]	158.6	n/a
RhB-PBP10 + MNP@Au	0.00956	0.003%
RhB-PBP10 + MNP@NH_2_	0.00486	0.002%
RhB-PBP10-Cys + MNP@Au	5.78400	3.65%
RhB-PBP10-Cys + MNP@NH_2_	7.91900	4.99%

The nanosystems were also characterized by FT-IR spectroscopy. Figure 1d summarizes the signals, showing the absorption peaks at ~ 550/cm which correspond to Fe–O stretching. The presence of peptide molecules on the MNP surface caused the characteristic stretching vibration for silane group to overlap with signals from peptides. The absorption peaks at 1650/cm are assigned to C=O bonds. The presence of aminoacids was detected also by peaks at around 1100 and 3200/cm which correspond to C–N stretching and N–H stretching respectively. Literature data indicate that the signal from S–H stretching bonds should appear near 2500/cm, however after immobilization to the MNP surface S–S bonds might form and the signal for this group can be registered around 550/cm [32]. In our case, this signal cannot be detected, because it is merged with the Fe–O stretching signal.

The zeta-potential and DLS studies were carried out for all nanosystems. Changes in the hydrodynamic diameter and zeta potential of modified nanoparticles with a comparison to bare (MNP@NH_2_, MNP@Au) were observed (Table 2). The zeta-potential measurements have shown that the addition of RhB-PBP10 peptide to magnetic nanoparticles decorated with a gold shell causes an increase of zeta-potential from − 18.7 to − 12.3 mV. Moreover, the addition of PBP10 peptide modified by cysteine and rhodamine converts zeta-potential to a positive value (from − 18.7 to + 12.9 mV), suggesting that this peptide interacts more strongly with the surface of nanoparticles than nonmodified PBP10 peptide. Nanoparticles with aminosilane shell act similarly to nanoparticles with a gold shell. After addition of PBP10 peptide, zeta-potential increases from − 11.8 to − 7.6 mV, while the addition of PBP10 modified by cysteine and rhodamine converts the signal of zeta-potential to positive values (from − 11.8 to 15.6 mV). The observed changes in zeta-potential suggest the interaction of peptides with nanoparticles surface. A decrease in hydrodynamic diameter of peptide-modified nanoparticles is also observed. According to Table 2, the average size of gold nanoparticles decreases about 100 and 200 nm after the addition of RhB-PBP10 and PBP10 modified by cysteine and rhodamine, respectively (from 442 nm to 346 and 256 nm, respectively). Similarly, average size changes of aminosilane-based nanosystems decrease from 965 nm (bare nanoparticles) to less than 100 nm (nanoparticles decorated with PBP10 peptide modified by cysteine and rhodamine).

**Table 2:** Hydrodynamic diameters and zeta potential values recorded for gold- and aminosilane-decorated nanoparticles modified by RhB-PBP10 and RhB-PBP10-Cys

	Hydrodynamic diameter (nm)	Zeta potential (mV)
MNP@Au	442	− 18.7 ± 0.9
RhB-PBP10 + MNP@Au	346	− 12.3 ± 0.8
RhB-PBP10-Cys + MNP@Au	256	12.9 ± 1.1
MNP@NH_2_	965	− 11.8 ± 0.4
RhB-PBP10 + MNP@NH_2_	834	− 7.6 ± 0.5
RhB-PBP10-Cys + MNP@NH_2_	98	15.6 ± 0.7

### Cytotoxicity of PBP10-containing nanosystems against human keratinocytes

**Fig. 2:**
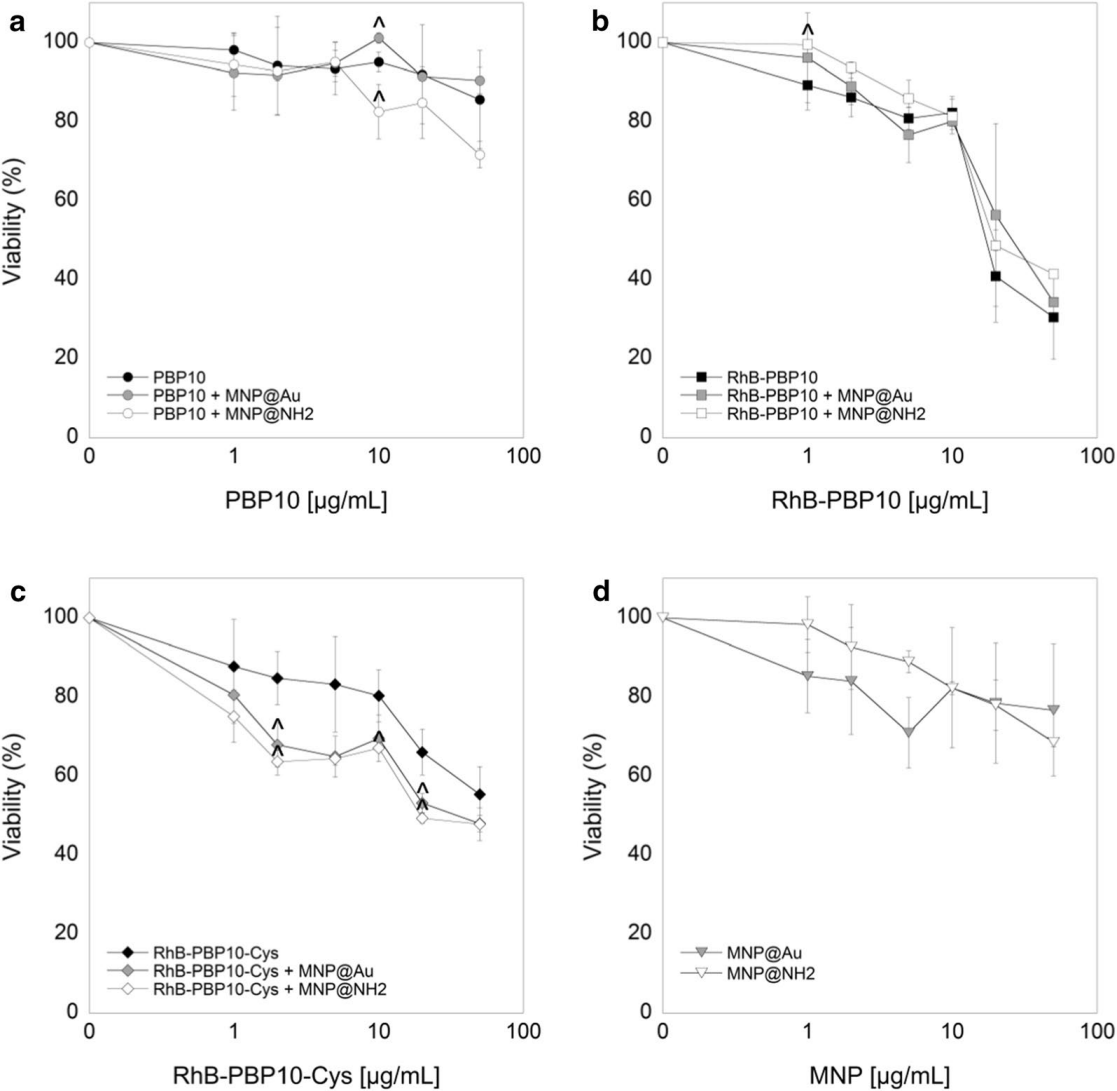
Biocompatibility of PBP10-based peptides and their magnetic derivatives. Metabolic activity of HaCaT cells treated with PBP10 (**a**), RhB-PBP10 (**b**) and RhB-PBP10-Cys (**c**) peptides in free form (black circles, squares and diamonds, respectively) and functionalized by gold- (+MNP@Au; grey symbols) and aminosilane-coated (+MNP@NH_2_; white symbols) magnetic nanoparticles for 24 h at doses ranging from 1 to 50 µg/mL. The viability of human keratinocytes incubated in the presence of bare MNP@Au (grey inverted triangles) and MNP@NH_2_ (white inverted triangles) is demonstrated in **d**. Results are presented as mean ± SD obtained from 3 experiments. ^^^Indicates statistically significant (p ≤ 0.05) activity of PBP10-containing nanosystems comparing to non-immobilized agents

In the first step of evaluation of potential immunomodulatory properties of PBP10-containing nanosystems, we performed a cytotoxicity assay for human keratinocytes. For this purpose, an MTT assay based on estimation of metabolic activity was employed. According to the results presented in the Fig. 2a–c, three forms of PBP10 peptides are characterized by relatively low toxicity against HaCaT cells at doses ranging from 1 to 10 µg/mL. The lowest toxicity was noted for PBP10 and its magnetic counterparts (i.e. PBP10 + MNP@Au, PBP10 + MNP@NH_2_) since 24 h incubation with these agents at a dose of 10 µg/mL resulted in viability of HaCaT cells in ranges from 101 ± 1.4% to 83 ± 6.9. Marginally higher cytotoxicity was noted for RhB-PBP10-Cys and its nanosystems (80 ± 6.6% for RhB-PBP10-Cys and 68 ± 0.6% for RhB-PBP10-Cys + MNP@NH_2_ for a dose of 10 µg/mL). The IC_50_ values for all tested gelsolin-related peptides were 98.9 ± 6.7 µg/mL, 30.3 ± 5.3 µg/mL and 59.3 ± 11.6 µg/mL for PBP10, RhB-PBP10, and RhB-PBP10-Cys, respectively, which indicates their low toxicity against mammalian cells. Importantly, in most cases immobilization of PBP10 peptides on the surface of magnetic nanoparticles does not increase the toxic effects of the peptides. Particularly, immobilization of peptide derivatives onto gold-decorated nanosystems seems to be particularly preferable, since the IC50 values for Au-based nanosystems are higher compared to free peptides (Table 3). We also observed relatively high biocompatibility for bare gold- and aminosilane-coated nanoparticles, considering that the number of HaCaT cells with lower metabolic activity did not exceed ~ 30% up to doses of 50 µg/mL (Fig. 2d). IC_50_ values for MNP@Au and MNP@NH_2_ were 74.9 ± 35.3 µg/mL and 85.0 ± 22.6 µg/mL, respectively. Additionally, up to a dose of 10 µg/mL no change in ROS generation in unstimulated HaCaT cells was noted (not shown data), which confirms that iron oxide-based nanostructures do not cause oxidative stress in our experimental settings. Based on these data, doses of 2, 5 and 10 µg/mL were chosen for further bacterial neutralization experiments.

**Table 3:** IC_50_ (the concentration that is required for 50% inhibition of the metabolic activity of cells) and MHC10% values (minimum hemolytic concentration that caused 10% hemolysis of human red blood cells) assessed for tested peptides and their magnetic derivatives

	IC_50_ (µg/mL)	MHC10% (µg/mL)
PBP10	98.85 ± 6.67	6.02 ± 2.72
PBP10 + MNP@Au	169.22 ± 55.66	58.97 ± 39.31
PBP10 + MNP@NH_2_	94.17 ± 19.19	852.86 ± 607.82
RhB-PBP10	30.31 ± 5.30	51.12 ± 6.31
RhB-PBP10 + MNP@Au	33.91 ± 1.41	105.17 ± 67.30
RhB-PBP10 + MNP@NH_2_	34.42 ± 1.61	10.19 ± 1.18
RhB-PBP10-Cys	59.25 ± 11.60	72.92 ± 25.95
RhB-PBP10-Cys + MNP@Au	42.24 ± 5.76	94.76 ± 42.32
RhB-PBP10-Cys + MNP@NH_2_	32.76 ± 10.14	56.02 ± 2.75
MNP@Au	74.90 ± 35.28	511.17 ± 102.47
MNP@NH_2_	85.12 ± 22.56	707.39 ± 644.03

Results are presented as the mean value calculated for 3 independent experiments ± SD

### Immobilization of PBP10 peptides on the surface of magnetic nanoparticles improves their hemocompatibility

In another set of experiments, we evaluated the toxicity of peptides and their magnetic derivatives using a red blood cell-based in vitro model. As demonstrated in Table 3, conjugation of GSN160-169 to rhodamine B, and supplementation of PBP10 with a terminal cysteine considerably decreased its hemolytic activity, considering that MHC10% for RhB-linked peptides rose from 6.0 ± 2.7 µg/mL to 51.1 ± 6.3 µg/mL and 72.9 ± 25.9 µg/mL for RhB-PBP10 and RhB-PBP10-Cys, respectively. Our previous studies showed significant reduction of hemolytic activity of membrane permeabilizing agents after their immobilization on the surface of iron oxide-based magnetic nanoparticles [33], and we recorded improved hemocompatibility of PBP10-derived nanosystems, particularly when gelsolin-related peptides were incorporated into gold-based nanostructures (increase from 1.3- to 9.8-fold when compared to unimmobilized agents; Table 3). Importantly, in the great majority of combinations, at a dose of 10 µg/mL, the release of hemoglobin from RBCs did not exceed 0.5–1%. The considerable differences between aminosilane- and gold-based nanosystems suggest that the type and molecular structure of nanomaterials intended for development of nanosystems is crucial in the design of therapeutic agents with satisfactory biocompatibility.

### PBP10 peptides and their magnetic derivatives exert bactericidal properties against both Gram-negative and Gram-positive bacteria

**Fig. 3:**
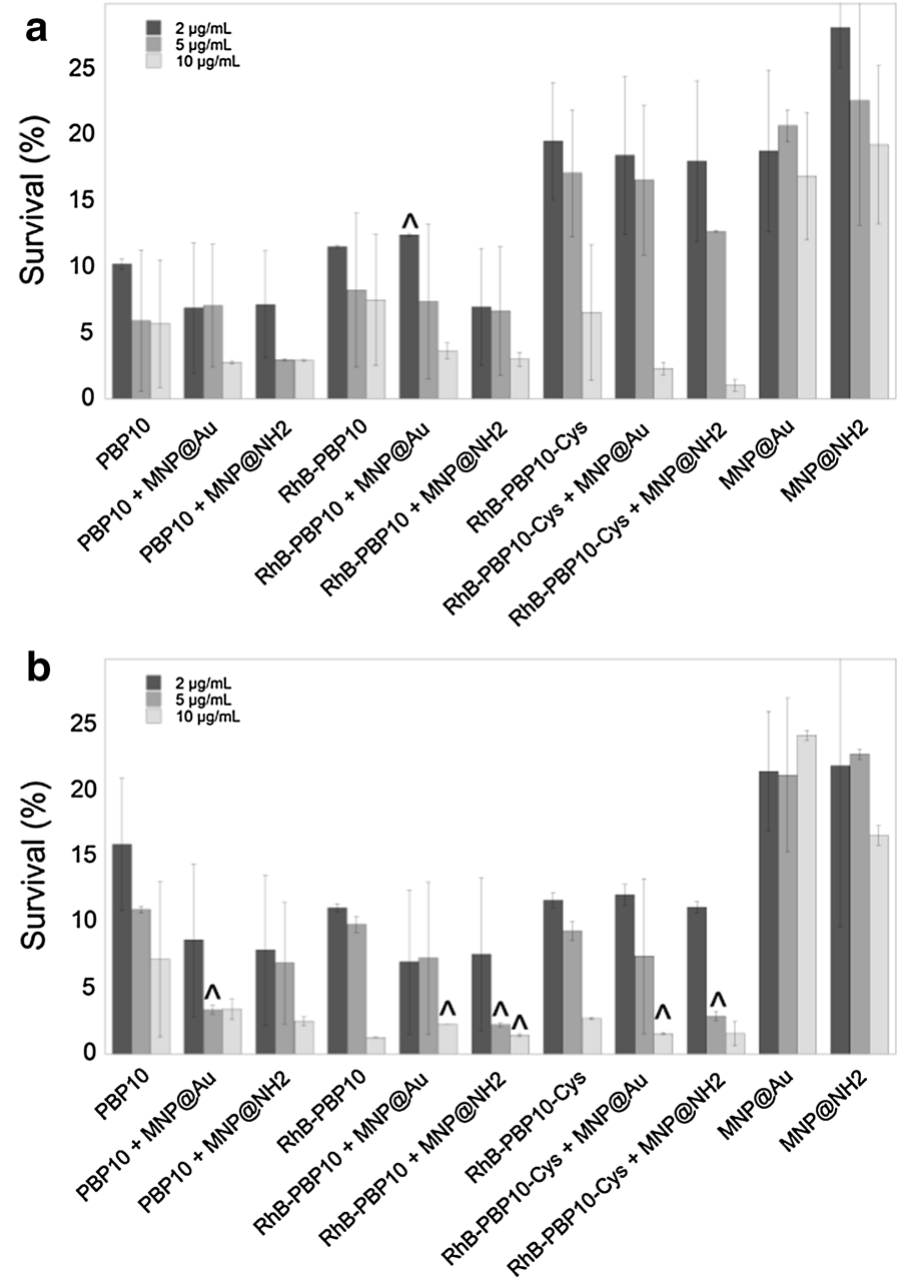
Antibacterial activity of PBP10-based nanosystems. The decrease of *E. coli* RS218 (**a**) and *S. aureus* A1 (**b**) strains survival incubated in the presence of indicated agents at concentrations of 2 µg/mL (dark grey columns), 5 µg/mL (grey columns) and 10 µg/mL (light grey columns) when compared to untreated bacterial samples (100%). Results are presented as mean ± SD obtained from 3 experiments. ^^^Indicates statistical significance when comparing MNP-based agents to non-immobilized peptides

In our previous research aimed to evaluate the antimicrobial activities of RhB-PBP10, we reported killing activity against the Gram-negative bacteria *E. coli* and *Pseudomonas aeruginosa* and the Gram-positive bacterium *Streptococcus pneumoniae* [34]. With this in mind, we investigated the bactericidal activity of gelsolin-derived peptides and their magnetic derivatives against *E. coli* RS218 and *S. aureus* A1. According to results presented in Fig. 3, both PBP10 and its rhodamine B-conjugated counterparts, RhB-PBP10 and RhB-PBP10-Cys, exert significant dose-dependent bactericidal effects against both Gram-negative and Gram-positive organisms resulting in a decrease of bacterial viability by more than 95%. In the case of *E. coli* (Fig. 3a), the addition of a terminal cysteine to the RhB-conjugated gelsolin sequence appears to have a small inhibitory effect on the peptide’s killing activity. In accordance with our previous research, the attachment of peptides to magnetic nanoparticles results in 1.7–6.4 fold increase of bactericidal activity.

### PBP10-based agents decrease the release of nitric oxide from stimulated keratinocytes

**Fig. 4:**
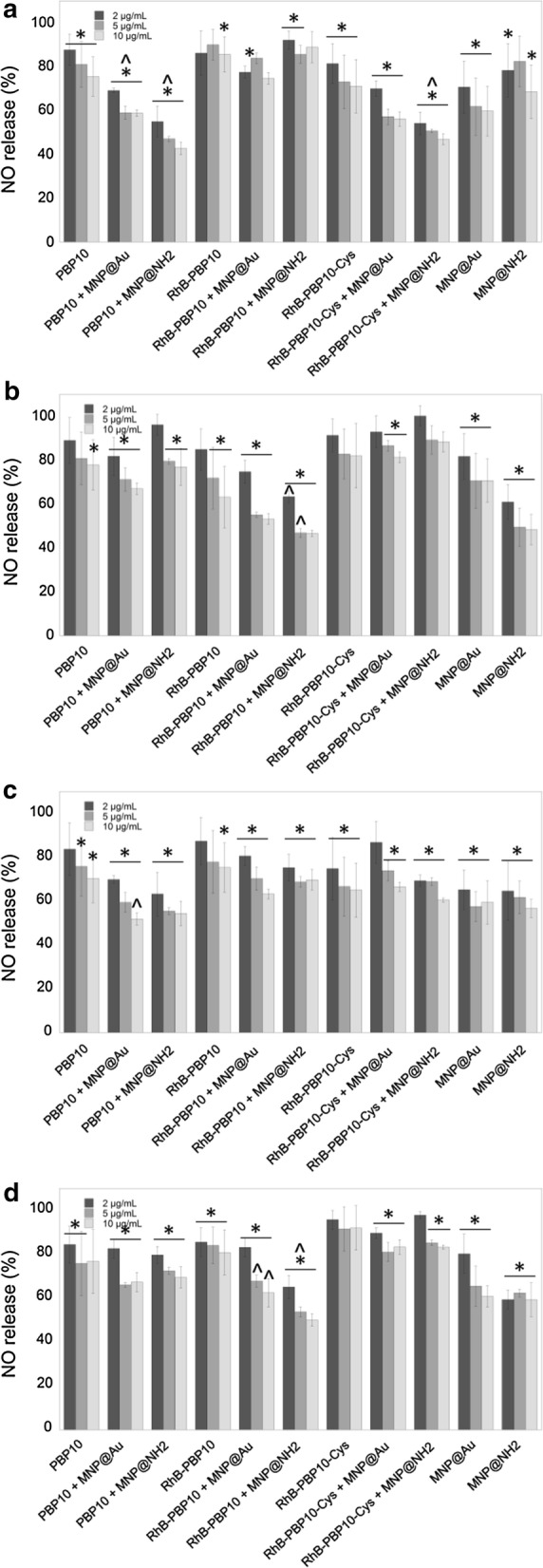
Reduction of nitric oxide (NO) release from stimulated human keratinocytes by PBP10-containing nanostructures. Release of NO from HaCaT cells stimulated with 1 µg/mL LPS (**a**), LTA (**b**) or heat-inactivated suspension of *E. coli* RS218 (**c**) or *S. aureus* A1 (**d**) and treated simultaneously for 24 h with indicated peptides and their magnetic derivatives at concentrations of 2 µg/mL (dark grey columns), 5 µg/mL (grey columns) and 10 µg/mL (light grey columns). Untreated stimulated control samples are indicated as 100%. Results are presented as mean ± SD obtained from 3 experiments. *Indicates statistically significant (p ≤ 0.05) activity of tested agents compared to untreated control samples, ^^^Indicates statistical significance when comparing MNP-based agents to non-immobilized peptides

Due to constant exposure of skin to environmental challenges, such as physical stress, trauma, chemical irritants, and infectious pathogens, keratinocytes constitute the first line of defense against external insults and participate in the innate immune response by secreting soluble inflammatory factors including NO and ROS. Therefore, inhibitors of NO and ROS production in stimulated keratinocytes might provide a valuable approach to limit inflammation [35]. With this in mind, we determined the nitrite level in the supernatants of HaCaT keratinocytes after 24 h exposure of cells to 1 µg/mL LPS, LTA or heat-killed bacterial suspensions, as an indicator of NO production, which is a well-documented response of human keratinocytes after inflammatory stimulation [35]. Intact heat-inactivated bacteria as infectious and inflammatory stimuli may better reflect a physiological encounter between keratinocytes and bacteria than the addition of purified LPS/LTA to cultures. As presented in the Fig. 4, treatment of stimulated HaCaT cells (indicated as 100%) with PBP10-derived agents at concentrations of 2, 5 and 10 µg/mL significantly limited the nitrite level in a dose-dependent manner. Interestingly, the anti-inflammatory effect of different forms of PBP10 peptide varied depending on the inflammatory stimulus. For LPS-treated cells (Fig. 4a), the highest activity was noted for PBP10 and its nanosystems (NO production was limited to 75.44 ± 8.97% for unmodified PBP10 [*p *= 0.090 when compared to untreated control] and to 42.76 ± 2.87% for PBP10 + MNP@NH_2_ at a dose of 10 µg/mL [p < 0.0001]), whereas RhB-PBP10 and its derivatives had the highest activity in LTA-stimulated samples (63.16 ± 14.01% for RhB-PBP10 alone [*p *= 0.0104] and 46.64 ± 1.51% for RhB-PBP10 + MNP@NH_2_ at a dose of 10 μg/mL [*p *< 0.0001]) (Fig. 4b). A similar tendency was observed when results of LPS- and LTA-treated cells (Fig. 4a, b) were compared with data obtained from samples incubated in the presence of heat-inactivated suspension of *E. coli* and *S. aureus,* respectively (Fig. 4c, d), which indicates that the activity of these agents depends strongly on the type of inflammatory stimulus. Functionalization of PBP10 using aminosilane-coated nanoparticles seems to be more efficient in augmentation of anti-inflammatory properties as measured by disruption of NO release (*p*-value ranging from 0.0039 to 0.0283 for PBP10 + MNP@NH_2_ and RhB-PBP10-Cys + MNP@NH_2_, respectively).

### PBP10-based agents protect cells from oxidative stress

**Fig. 5:**
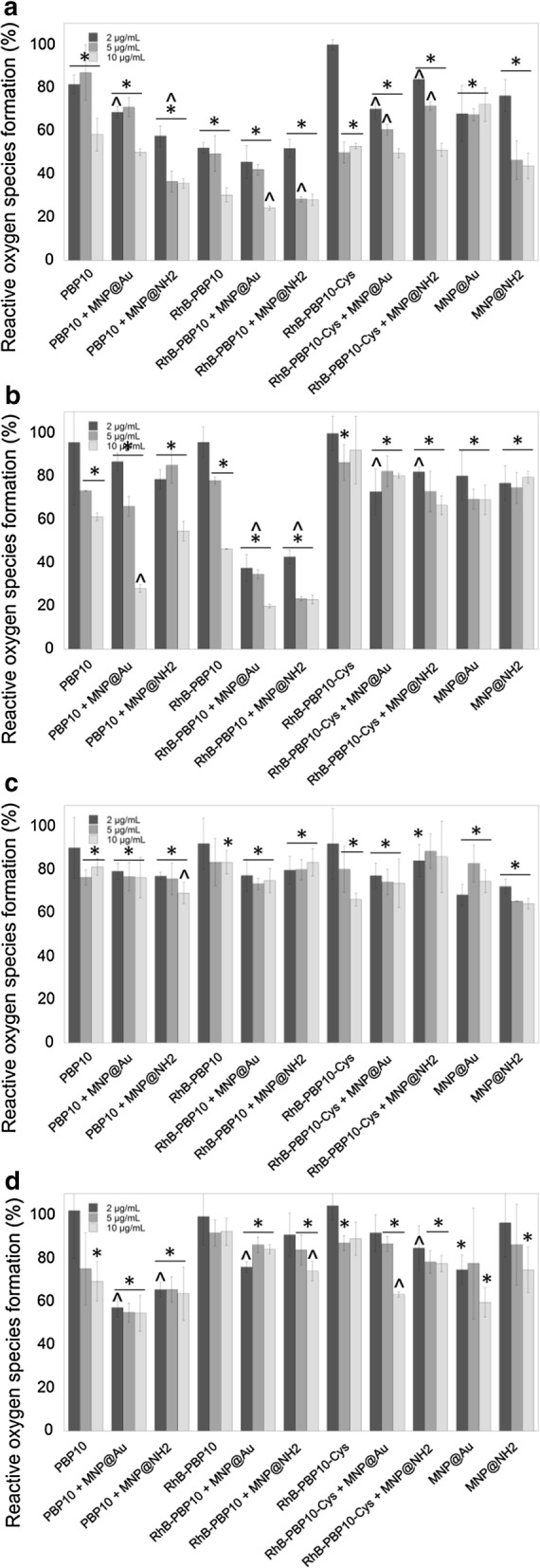
Inhibition of reactive oxygen species (ROS) generation in stimulated HaCaT cells. Level of reactive oxygen species formation in HaCaT cells stimulated with 1 µg/mL of LPS (**a**), LTA (**b**) or heat-inactivated suspension of *E. coli* RS218 (**c**) or *S. aureus* A1 (**d**) and treated simultaneously for 24 h with PBP10-based peptides and their magnetic derivatives at concentrations of 2 µg/mL (dark grey columns), 5 µg/mL (grey columns) and 10 µg/mL (light grey columns). Untreated stimulated control samples are indicated as 100%. Results are presented as mean ± SD obtained from 3 experiments. *Indicates statistically significant (p ≤ 0.05) activity of tested agents compared to untreated control samples, ^^^indicates statistical significance when comparing to MNP-based agents to non-immobilized peptides

Together with the promising results of NO release assays, significant inhibition of ROS generation in stimulated keratinocytes was noted (Fig. 5). Dysregulated ROS formation is involved in the pathogenesis of various inflammatory conditions, and agents with antioxidant properties might be considered to be potential therapeutic agents for ROS-mediated inflammatory skin diseases [36]. To study the effect of PBP10-based nanosystems on the inflammatory response of HaCaT cells, we measured ROS production after stimulation with LPS, LTA, and bacterial suspensions. According to data presented in Fig. 5, PBP10-based nanosystems strongly inhibit the inflammatory stimulus-induced ROS synthesis. Interestingly, in contrast to NO release assay, the highest activity was noted for RhB-PBP10 and its nanosystems, suggesting that intracellular localization of therapeutic agents might be crucial for effective ROS production limitation. According to the data in Fig. 5a, for LPS-stimulated samples ROS production was limited from 30.33 ± 3.31% for unmodified RhB-PBP10 [p < 0.0001] to 24.27 ± 0.94% for RhB-PBP10 + MNP@Au at a dose of 10 µg/mL [p < 0.0001]); inhibition of ROS synthesis in LTA-treated cells was 46.42 ± 0.19% and 19.97 ± 0.77% for RhB-PBP10 and RhB-PBP10 + MNP@Au, respectively [*p *< 0.0001 in both cases] (Fig. 5b). Most of the PBP10 derivatives, both in free and immobilized forms inhibited intracellular ROS generation when compared to untreated stimulated controls, which highlights their potential as anti-oxidative agents.

### The release of IL-8 from stimulated keratinocytes is decreased by PBP10-derived nanoagents

**Fig. 6:**
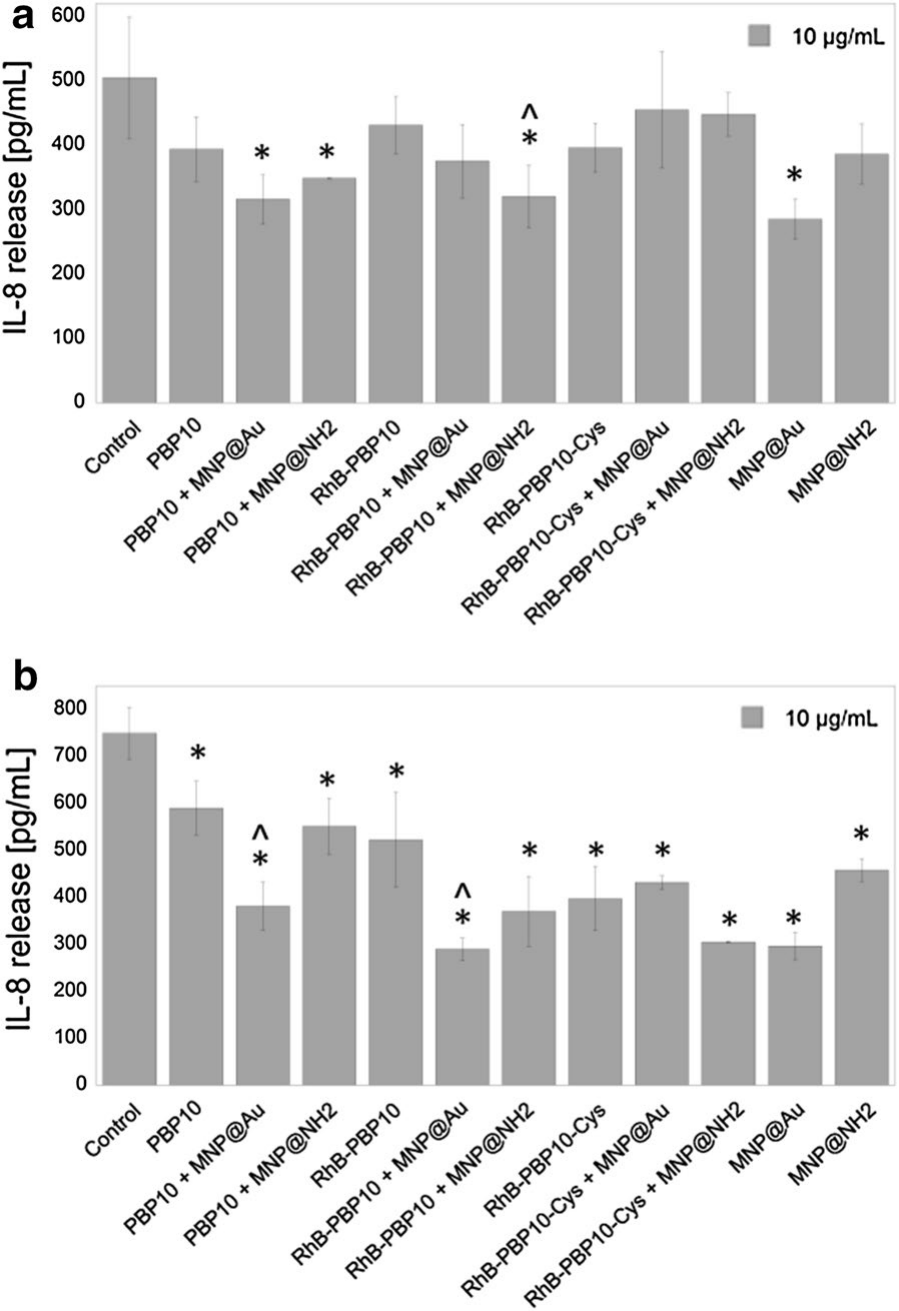
Immunomodulatory properties of PBP10-containing nanosystems. The release of IL-8 from LPS- (**a**) and LTA-stimulated (**b**) HaCaT cells after treatment with PBP10 peptides in free form and immobilized on magnetic nanoparticles. Results are presented as mean ± SD obtained from 3 experiments. *Indicates statistically significant (p ≤ 0.05) activity of tested agents compared to untreated control samples stimulated with LPS/LTA, ^^^indicates statistical significance when comparing MNP-based nanosystems to non-immobilized peptides

The release of IL-8 is implicated in the pathogenesis of inflammatory-related diseases and is highly expressed by keratinocytes [37]. As presented in Fig. 6a, b, IL-8 levels in untreated LPS- and LTA-stimulated keratinocytes were 504.9 ± 49.39 pg/mL and 749.2 ± 55.81 pg/mL, respectively. In contrast, treatment of cells with a set of PBP10 peptides resulted in a decrease of IL-8 release to the level of 393.5–431 pg/mL in LPS-stimulated samples and to 397.5–589.7 pg/mL in LTA-treated cells. Further decrease of IL-8 release was also observed after functionalization of peptides by magnetic nanoparticles, particularly by MNP@Au. These data indicate that nanosystems have stronger anti-inflammatory properties than their non-modified counterparts, but this effect depends on the molecular properties of the nanostructure, as indicated by the relatively low impact of functionalization for RhB-PBP10-Cys.

### Treatment with PBP10 and its derivatives prevents LPS-induced changes in biomechanical properties of cells

**Fig. 7:**
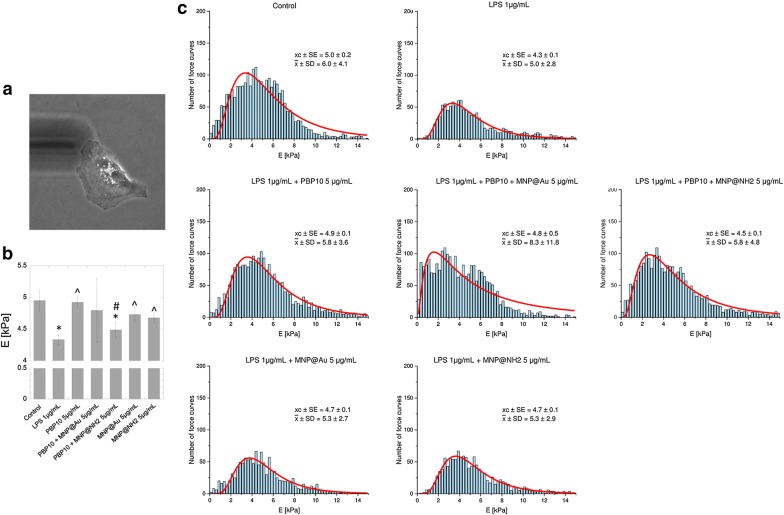
Changes in nanomechanical properties of LPS-stimulated cells determined by AFM using indentation depth value of 300 nm. Representative photograph of a single A549 cell probed using AFM working in force spectroscopy mode in liquid conditions (**a**). Summary of Young’s modulus analysis for cells treated with indicated agents at a dose of 5 µg/mL (**b**). Histograms presenting the distribution of force curves obtained from unstimulated cells, LPS-treated cells and endotoxin-stimulated cells incubated in the presence of PBP10 peptide and its magnetic derivatives are demonstrated in **c**. Results are presented as mean ± SD. * and ^^^indicate statistically significant (p ≤ 0.05) activity of tested agents compared to untreated control samples and LPS-stimulated cells, respectively. ^#^Indicates statistical significance when comparing MNP-based agents to the non-immobilized peptide

**Fig. 8:**
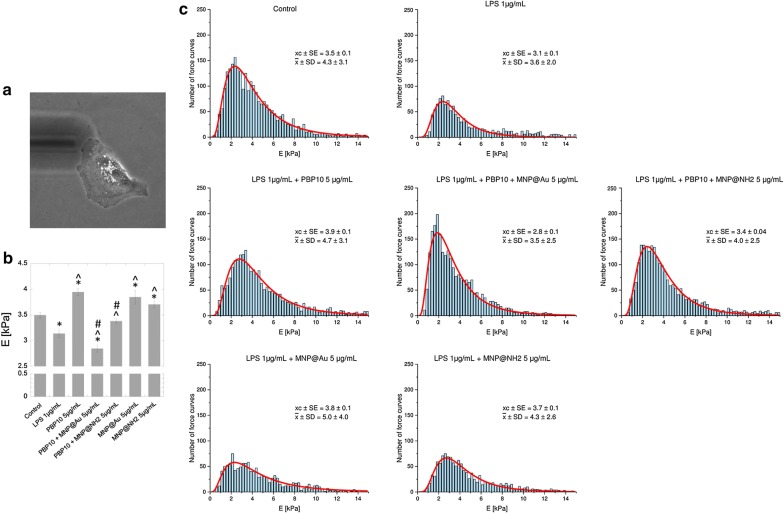
Changes in nanomechanical properties of LPS-stimulated cells determined by AFM using an indentation depth value of 1 µm. Representative photograph of a single A549 cell probed using AFM working in force spectroscopy mode in liquid conditions (**a**). Summary of Young’s modulus analysis for cells treated with indicated agents at a dose of 5 µg/mL (**b**). Histograms presenting the distribution of force curves obtained from unstimulated cells, LPS-treated cells and endotoxin-stimulated cells incubated in the presence of PBP10 peptide and its magnetic derivatives are demonstrated in **c**. Results are presented as mean ± SD. * and ^^^Indicate statistically significant (p ≤ 0.05) activity of tested agents compared to untreated control samples and LPS-stimulated cells, respectively. ^#^Indicates statistical significance when comparing MNP-based agents to the non-immobilized peptide

Atomic force microscopy (AFM) permits studies of cellular morphology and mechanical properties of the cell surface at the nanoscale. Considering previous reports demonstrating (i) alterations of biomechanical properties of cells in a variety of physiological and pathological processes [38] and (ii) the possibility to employ AFM-based analyses to evaluate anti-inflammatory drugs in LPS-activated cell models [38, 39], we investigated changes in HaCaT cell stiffness upon exposure to LPS and subsequent treatment with PBP10 and its magnetic derivatives. We operated AFM force spectroscopy to assess cell cortical stiffness (Young’s modulus) using two indentation depths: 300 nm (Fig. 7) and 1 µm (Fig. 8). According to the statistical analysis presented in Fig. 7b, the Young’s modulus of the cell cortex dropped from 5.0 ± 0.2 kPa for control cells to 4.3 ± 0.07 kPa for 1 µg/mL LPS stimulated cells (*p*-value = 0.0176), which indicated that lung epithelial cells became softer after 24-hour LPS stimulation. The same conclusion was drawn when AFM analysis using an indentation depth of 1 µm was performed (Fig. 8b); upon exposure to bacterial endotoxin cell stiffness was decreased from 3.5 ± 0.06 kPa for control cells to 3.1 ± 0.08 kPa for LPS-treated cells (*p *= 0.0003). In contrast, simultaneous treatment of cells with PBP10 in free and immobilized form resulted in the reverse of the LPS effect and a partial increase in cell stiffness. A particularly visible effect was noted for unmodified PBP10; for both indentation depths, treatment of cells with 5 µg/mL PBP10 increased Young’s moduli to 4.9 ± 0.1 kPa (*p *= 0.0001) and 3.9 ± 0.1 kPa (*p* < 0.0001), respectively (Figs. 7b, 8b), which was comparable with values recorded for unstimulated control cells. Statistical significance, when compared to LPS-treated samples, was also observed for bare magnetic nanoparticles, indicating their potential for neutralization of LPS-induced effects.

## Discussion

Even during treatment with conventional antibiotics, bacteria can release endotoxins like LPS or other pathogenicity factors from their cell envelope that activate Toll-like receptors (TLRs) and induce strong inflammatory reactions [40]. Therefore, endotoxin-neutralizing agents, specifically developed to neutralize these pathogenicity factors and with potent ability to abrogate TLR-mediated inflammatory responses, represent an innovative approach for the treatment of bacterial infections. To date, some natural and synthetic agents with both antibacterial and LPS-binding and neutralizing activity, mostly from the group of cationic antimicrobial peptides (AMPs), were presented as potential candidates for treatment of microbial-related diseases, including sepsis, LPS-induced inflammation during cystic fibrosis lung disease and skin infections [6, 41, 42]. In this study, we employed a set of gelsolin-related peptides, derived from the PIP2-binding site of human gelsolin to develop MNP-based nanosystems able to bind and neutralize LPS and LTA. Previous studies, using transgenic gelsolin-lacking mouse models, strongly indicated the beneficial effects of gelsolin and gelsolin-derived compounds in maintaining proper functions of the immune system. Witke et al. revealed that gelsolin-null mice exhibited a number of dysfunctions in inflammatory reactions, including delayed in vivo migration of neutrophils into peritoneal exudates and impaired chemotaxis [43]. Exogenous gelsolin was also found to limit neutrophil migration, scavenge soluble pro-inflammatory mediators, and inhibit neutrophil adhesion to endothelial surfaces in a mouse model of acute lung injury [9, 44]. Extracellular gelsolin also has a beneficial activity on macrophage-mediated antimicrobial and host defense functions through a nitric oxide synthase type III (NOS3)-dependent mechanism and improved bacterial binding and killing [45, 46]. These studies highlight the potential of extracellular gelsolin in the treatment of infections and microbial-associated medical conditions. Anti-inflammatory activity was also strongly suggested for gelsolin-derived peptide based on the PIP2-binding sequence of gelsolin [8, 9, 14, 34]. With this in mind, we designed peptide-based nanosystems, consisting of GSN160-169-derived peptides as bioactive compounds and magnetic nanoparticles as highly biocompatible nanocarriers (Fig. 1a). For this purpose, PBP10 peptides were attached to gold- and aminosilane-decorated nanoparticles via chemisorption of thiol groups to gold surface and electrostatic interaction with aminosilane shells, respectively, as confirmed by fluorescence analysis (Fig. 1c) and FT-IR spectroscopy (Fig. 1d).

Using gold- and aminosilane-coated iron oxide-based magnetic nanoparticles as nanocarriers and compounds with additional anti-inflammatory and antimicrobial activity, we have been able to modulate the production of pro-inflammatory soluble factors, including NO, ROS, and IL-8 from cells after stimulation with purified LPS/LTA and heat-killed bacteria, *E. coli* and *S. aureus*. Several previous observations indicated that metal and metal oxide nanoparticles possess a high therapeutic value relating to its anti-inflammatory and bactericidal characteristics. Due to their interaction with cell wall components, ROS generating-properties and ability to disrupt metabolic pathways, several metal oxide-based nanoparticles have been proposed to be used in limiting microbial infections [47]. Recently, magnetic nanoparticles with aminosilane shells were successfully employed as factors modulating the anti-inflammatory activity of 1,4-DHPs [25]. Prasad et al. demonstrated the utility of nanosystems composed of long C18 acyl chains tethered to Fe_3_O_4_/Au/Fe_3_O_4_ nanoflowers for simultaneous removal and detection of endotoxins from food to pharmaceutical preparations due to reversible binding with the bioactive lipid A component present on the LPS molecule [21]. Administration of gold nanoparticles (AuNPs) was found to decrease LPS-induced eye inflammatory response in a rat model of endotoxin-induced uveitis, which is determined by a decrease in TLR4 content and NF-κB activation [23]. Results from mouse models showing protection against a lethal inhalation challenge of *Burkholderia mallei* using a gold nanoparticle-linked glycoconjugate vaccine are also promising [24]. However, the effects of AuNP in vivo after repeated administration are still unclear and contradictory. Polyethylene glycol-coated AuNPs were shown to activate p38 MAPK/NF-κB pathways in RAW264.7 cells, which suggests potential immunotoxicity of these compounds due to their ability to stimulate macrophages to release aberrant or excessive pro-inflammatory mediators [48]. On the other hand, Ma et al. and Nishanth et al. indicated suppression of LPS-induced activation of the NF-κB signaling pathway in RAW264.7 cells and high biocompatibility of gold nanoparticles, with small inflammatory reactions noted only after prolonged exposure [19, 49]. Our preliminary studies have shown that neither MNP@NH_2_ nor MNP@Au induces inflammatory effects in mammalian cells, and they do not affect NO synthesis or IL-8 release in a broad spectrum of doses and increase slightly ROS generation only at a high dose of 50 µg/mL (not shown data). In contrast, we show that at doses that are reported as safe for the culture of human keratinocytes (i.e. 2–10 µg/mL) (Fig. 2), a majority of nanoparticle-based combinations exert efficient inhibition of LPS- and LTA-induced cellular effects, serving as protective agents during microbial-associated inflammation. Importantly, immobilization of PBP10 peptides on the surface of magnetic nanoparticles does not increase their toxic effects against mammalian cells (Fig. 2a–d) and provides a possibility to significantly improve the hemocompatibility of gelsolin-derived peptides (Table 3), which is in agreement with our previous research demonstrating a decrease of hemolytic activity of membrane-active agents after their immobilization on the surface of iron oxide-based nanoparticles [50]. All tested combinations are characterized by relatively low toxicity against mammalian cells, particularly in comparison with melittin, an amphipathic peptide from the group of AMPs with potent antibacterial, antiviral, and anti-inflammatory activities, proposed as a therapeutic agent with the ability to prevent *Propionibacterium acnes*-induced inflammatory skin diseases [51]. With regard to hemolytic activity, it is noteworthy that PBP10-based agents are able to lyse RBCs, but their lytic concentrations are significantly higher than those required for bacterium-killing and immunomodulatory activity. This strongly supports the potential of these combinations in the treatment of both topical and systemic infections, as suggested before for endogenous and synthetic AMPs such as LL-37, RR, and RRIKA [52, 53]. RhB-PBP10 is structurally similar to AMPs due to its cationic charge, a short sequence, ability to cross membranes and amphipathic chemical character [14]. Similar to other antimicrobial peptides, including human cathelicidin-derived LL-37, RhB-PBP10 inhibits the growth of Gram-positive and Gram-negative microorganisms, including *Escherichia coli¸ Pseudomonas aeruginosa, Streptococcus pneumoniae* and *Bacillus subtilis,* which results from penetration of the peptide into the membrane bilayer and membrane destabilization [14, 34]. In agreement with these reports, we show a significant bactericidal activity of PBP10-related peptides and their magnetic nanoparticle-based nanosystems against *Escherichia coli* and *Staphylococcus aureus* (Fig. 3a, b, respectively), despite the lack of phosphoinositides (i.e. the presumed native ligand for gelsolin-related peptides) in these microorganisms. Although rhodamine B linkage to unmodified PBP10 was reported previously to be essential for the antibacterial function of RhB-PBP10 [34], we did not detect significant differences in killing activity of PBP10 and its RhB-conjugated counterpart, which is likely related to different experimental settings and different bacterial strains. More importantly, we recorded a slight decrease of antibacterial activity of PBP10, particularly against *E. coli* suspension, when the peptide sequence was supplemented with an additional C-terminal cysteine (RhB-PBP10-Cys) in order to improve the attachment of the peptide to gold-decorated magnetic nanoparticles (Fig. 3a). Similar observations were noted when the activity of RhB-PBP10-Cys was tested against fungal cells and other bacterial strains (data not shown).

Although a continuous and rapid development of new diagnostic and therapeutic methods, applicable in the detection and therapy of bacterial infections is observed, it is necessary to continue to search for new antimicrobial factors with high selectivity [40, 54]. Despite the promising results, some evidence suggests that the employment of AMPs and their derivatives in the treatment of topical and systemic infections might be limited due to rather a poor selectivity for bacterial over mammalian cells, which results from the unspecific membrane-permeabilizing mechanism of microbial killing characterized for AMPs. We show that gelsolin-derived peptides exert a statistically significant growth inhibitory effect against clinically relevant bacterial strains, whereas cytotoxicity against human keratinocytes culture was not observed under employed conditions and concentrations. Previous studies suggest that higher specificity of presented gelsolin-related peptides for targeting bacteria rather than host cells is determined by the asymmetrical distribution of phospholipids in the external membranes of eukaryotic cells [14]. Another explanation of this phenomenon assumes that LPS and LTA present in external bacterial membranes are bacterial targets for PBP10 peptides and their magnetic derivatives, attracting specifically these agents and increasing peptide adsorption to the bacterial surface [55, 56]. Moreover, the possibility that LPS and LTA in association with other bacterial-wall molecules form binding sites for AMPs cannot be ruled out [55].

From a variety of molecular mechanisms mediating the inflammatory responses, production of pro-inflammatory cytokines and inflammatory molecules, such as iNOS-derived NO and reactive oxygen species seems to be the most prominent. Due to a number of virulence factors, including a broad spectrum of proteins and proteases with cytotoxic activity, phenol-soluble modulins and heat-shock proteins, both *S. aureus* and *E. coli* induce strong inflammatory response in human keratinocytes due to their recognition by TLR receptors, particularly TRL-2 and TLR-4, whose expression is strongly regulated by microbial components [57–59]. Nitric oxide is recognized as an important cellular modulator and signaling molecule, produced by a variety of human cells, including keratinocytes and macrophages during inflammatory reactions in the skin, where NO mediates cytotoxicity, controls bacterial infection and acts as an immunoregulatory factor [35]. As NO is synthetized primarily by constitutively expressed isoforms of NO synthases present in skin, a significant amount of keratinocyte-derived NO comes from the inducible isoform of this enzyme (iNOS), whose expression is increased after challenge with bacterial endotoxins, cytokines (e.g. IL-1β, IFN-γ, TNF-α, IL-8) and neuropeptides [60]. Importantly, overexpression of iNOS and the resulting augmentation of NO generation was demonstrated in some inflammatory-based and autoimmune-related skin diseases, such as psoriasis, sunburn erythema and edema [61]. Despite the fact that a compelling number of studies demonstrated that NO generation by skin cells is a crucial component of innate immunity serving as protective agent against several pathogens including *Mycobacterium leprae, Leishmania spp., E. coli,* and *Candida albicans,* excessive production of nitric oxide can lead to edema, prolonged inflammation and injury by promoting the infiltration of macrophages and lymphocytes into the tissue [35]. In effect, recognition of the pleiotropic biological activity of NO in a variety of medical conditions has resulted in the development of therapeutic approaches aimed to modulate NO production and a few pharmacological agents that both release NO and limit its production have been described [35, 62, 63]. It was confirmed that inhibition of the inducible NO pathway in human keratinocytes is partially involved in therapeutic effects of retinoic acid derivatives [64] and was also presented to be valuable to relieve symptoms of flushing and erythema in the skin [61].

In addition to reports demonstrating the anti-inflammatory features of agents limiting the NO generation in some medical conditions, antioxidants have been proposed to be beneficial in oxidative stress-induced inflammation. To date, reactive oxygen species released from inflammatory cells into the extracellular compartments and causing local propagation of the inflammatory reaction and tissue damage are recognized as one of the critical factors in inflammatory skin diseases [65]. Importantly, reactive oxygen intermediates interact with NO to generate a second line of reactive molecules that can attack a number of nucleophilic extracellular and intracellular targets. Previously, superoxide anions have been reported to react with NO to form peroxynitrite, a potent inducer of lipid and protein peroxidation, tyrosine nitration and other free radical-mediated reactions in *Propionibacterium acnes* dependent skin infection [66]. Similarly, keratinocytes exposed to UVB or arsenite produce both superoxide anions and NO, potentially leading to the peroxinitrite formation and thus DNA and protein damage in keratinocyte cultures [67]. Considering the implication of TLR2-mediated pathway in *P. acnes*-induced acne and reports demonstrating the significance of this receptor in staphylococcal infections, we hypothesize that similar ROS- and peroxynitrite-associated mechanism can mediate the apoptosis of bacterial-stimulated keratinocytes in our experimental model (data not shown). Therefore, the inhibition of ROS production or scavenging the released ROS may be important in preventing excess tissue damage during skin infection and inflammation [65]. With this in mind, we show that upon treatment of endotoxin- and bacterial-stimulated keratinocytes with PBP10-based nanosystems both NO (Fig. 4) and ROS (Fig. 5) release are significantly reduced. It cannot be ruled out that this limited effect is partially conditioned by keratinocyte death, but since ROS and NO production was inhibited to a significantly stronger degree, than results from cytotoxicity measurements (for instance to ~ 20% for RhB-PBP10 + MNP@Au when cytotoxicity did not excess 30% depending on the tested agent) we suggest that this effect has to be conditioned by immunomodulatory properties of the nanosystems. This effect seems to be stimuli-dependent. We hypothesize that the activities of nanosystems between LPS- and LTA-treated samples are determined by differences in binding, and thus neutralization of these pathogenic factors by gelsolin-derived nanosystems. Additionally, despite the fact that the tendency in the detected neutralizing activity of PBP10-containing nanosystems does not significantly fluctuate between cells treated with purified LPS/LTA and heat-inactivated bacteria, we observed some alternations in biological activity in bacterial *versus* extract-treated samples. We suggest that slight differences between these pairs might be conditioned by additional features, including bactericidal and membrane-permeabilizing properties and other bacterium-associated factors, such as a wider spectrum of microbial-derived stimulatory compounds present on the surface of microorganisms, which are able to stimulate TLRs and thus, affect the generation of these soluble mediators in HaCaT cells and induce inflammatory responses [59]. This issue needs to be thoroughly investigated.

In correlation with results presenting a statistically significant suppression of NO and ROS generation, a decrease in the amount of IL-8 detected in the extracellular environment was observed (Fig. 6). Because psoriatic keratinocytes have previously been shown to over-express mRNA transcripts for iNOS in the response to IL-8 [68] and cultured keratinocytes have been reported to produce a large amount of IL-8 during TLR2-mediated *P. acnes* skin infection [66], the detection of a simultaneous decrease of all inflammatory-associated mediators, i.e. nitric oxide, ROS, and IL-8 seems to be reasonable. Moreover, similarly to the results of NO generation assay (Fig. 4) we observed stimuli-dependent effects of PBP10-containing agents—PBP10 and RhB-PBP10 and their nanosystems seemed to be most effective in the limitation of IL-8 release in LPS- and LTA-treated cells, respectively (Fig. 6a, b), which highlights the link between nitric oxide and IL-8 generation and release.

One of the newer analytic approaches aimed to evaluate the anti-inflammatory drugs in LPS-activated cell culture-based models is atomic force microscopy, a high resolution imaging technique permitting to observe and analyze biological samples under physiological conditions using nanoscale resolution at the single cell level [69]. Recent studies indicate that changes in nanomechanical properties of cells reflect the transformation of cell physiology during the processes of molecule presentation and recognition, signal sensing, and increased expression of surface molecules or activation of cells. The scale of this phenomenon might be quantitatively described using the relative value of the Young’s modulus [70]. Importantly, the reverse of stimuli-induced cellular effects might be recognized as an indicator of biological activity of some drugs [39]. Previously, AFM was successfully used as an analytic tool to detect LPS-induced inflammatory responses in macrophages and monocytic cell cultures [38, 39], to analyze mechanisms of pathophysiological neutrophil mechanics in endotoxemia-related inflammatory conditions [71] and to assess the reduction of stiffness-dependent exacerbation of inflammatory processes [72, 73]. In our study, we used AFM to investigate whether a set of PBP10 peptides and their magnetic derivatives possess the potential to inhibit LPS-mediated alterations in nanomechanical properties of endothelial cells, and we found that endotoxin-induced decrease of cellular stiffness might be effectively turned back using PBP10 and its magnetic counterparts. To better understand the mechanism of PBP10-mediated effects we performed AFM elasticity measurements using two indentation depths: 300 nm and 1 µm, which allowed to assess local and average effect for the whole analyzed cell, respectively. In accordance with previous studies indicating the non-homogeneity of the cytoskeleton and pointing out the differences between the mechanical properties of cell’s surface and deeper parts of a cell [69], we recorded higher Young’s modulus values for smaller indentation depths, which corresponds to stiffness of regions rich in the network of actin filaments (Fig. 7b) and lower relative values of this parameter, when overall stiffness of the cell was analyzed (Fig. 8b) [69]. Importantly, we observed statistically significant changes in Young’s modulus of LPS-treated cells, which indicates alterations in actin reorganization upon endotoxin stimulation [39]. Moreover, incubation of LPS-treated cells with PBP10 peptide and MNP@Au/MNP@NH_2_ resulted in an increase of cellular stiffness to values comparable with non-stimulated control samples, which is most likely related with LPS binding and neutralizing properties of PBP10 peptide and nanoparticles. Pi et al. in their research aimed to evaluate the anti-inflammatory activity of dexamethasone and quercetin in a macrophage-based in vitro model and demonstrated that anti-LPS effects of these compounds are determined by the decreased binding of LPS and CD14 receptor on the surface of RAW264.7 [39]. We hypothesized that a similar phenomenon might occur in our experimental settings, nevertheless more complex single cell-based analyses are required to state this. An interesting observation noted during this study was a statistically significant PBP10-mediated increase of cellular stiffness when large indentation depth analysis was performed, which indicates that treatment with this peptide raises not the only stiffness of cell membrane, but also the whole cell interior (Fig. 8b, c). Based on our previous report, indicating an increase in stiffness and F-actin content in the cortical region of lung epithelial cells upon treatment with LL-37 peptide and accompanied by a decrease in transepithelial permeability and *Pseudomonas aeruginosa* uptake [73], we suggest that stiffening of cells in the response to treatment with PBP10-containing agents, both in free and immobilized form might provide an additional protective mechanism of these agents in microbial-associated medical conditions.

## Conclusions

The experiments described here demonstrate that PBP10, PBP10-derived peptides and nanosystems consisting of iron oxide-based magnetic nanoparticles coupled to these compounds might be useful in the neutralization of bacterial pathogenicity factors, including LPS and LTA and thus, can be successfully employed in the modern therapy of microbial-associated conditions, including drug resistant infections and bacteria-induced inflammatory states. Importantly, the attachment LPS/LTA-binding peptides to the surface of MNPs obtained via electrostatic interaction/chemisorption augments the anti-inflammatory and bactericidal capability of gelsolin-derived peptides with subsequent improvement of their cyto- and hemocompatibility, which highlights the potential of nanotechnology-based approaches. Since the severity of inflammatory states is conditioned by the balance between inflammatory and anti-inflammatory factors, inhibiting the release of nitric oxide, ROS, and IL-8 and reversing endotoxin-induced cellular effects using appropriate antioxidants and anti-inflammatory molecules could be considered as a potential treatment of SSTIs and sepsis.
